# Hyperresponsiveness to inhaled but not intravenous methacholine during acute respiratory syncytial virus infection in mice

**DOI:** 10.1186/1465-9921-6-142

**Published:** 2005-12-05

**Authors:** Rachel A Collins, Rosa C Gualano, Graeme R Zosky, Constance L Atkins, Debra J Turner, Giuseppe N Colasurdo, Peter D Sly

**Affiliations:** 1 Division of Clinical Sciences, Telethon Institute for Child Health Research, Centre for Child Health Research, The University of Western Australia, PO Box 855, West Perth WA 6872, Australia; 2 Department of Pharmacology, Co-Operative Research Centre (CRC) for Chronic Inflammatory Diseases, University of Melbourne, Parkville, Victoria, Australia; 3 Department of Pediatrics, University of Texas Health Science Center – Houston, Texas, USA

**Keywords:** forced oscillation, airway resistance, physiology

## Abstract

**Background:**

To characterise the acute physiological and inflammatory changes induced by low-dose RSV infection in mice.

**Methods:**

BALB/c mice were infected as adults (8 wk) or weanlings (3 wk) with 1 × 10^5 ^pfu of RSV A2 or vehicle (intranasal, 30 μl). Inflammation, cytokines and inflammatory markers in bronchoalveolar lavage fluid (BALF) and airway and tissue responses to inhaled methacholine (MCh; 0.001 – 30 mg/ml) were measured 5, 7, 10 and 21 days post infection. Responsiveness to iv MCh (6 – 96 μg/min/kg) in vivo and to electrical field stimulation (EFS) and MCh in vitro were measured at 7 d. Epithelial permeability was measured by Evans Blue dye leakage into BALF at 7 d. Respiratory mechanics were measured using low frequency forced oscillation in tracheostomised and ventilated (450 bpm, *f*lexiVent) mice. Low frequency impedance spectra were calculated (0.5 – 20 Hz) and a model, consisting of an airway compartment [airway resistance (Raw) and inertance (Iaw)] and a constant-phase tissue compartment [coefficients of tissue damping (G) and elastance (H)] was fitted to the data.

**Results:**

Inflammation in adult mouse BALF peaked at 7 d (RSV 15.6 (4.7 SE) vs. control 3.7 (0.7) × 10^4 ^cells/ml; p < 0.001), resolving by 21 d, with no increase in weanlings at any timepoint. RSV-infected mice were hyperresponsive to aerosolised MCh at 5 and 7 d (PC_200 _Raw adults: RSV 0.02 (0.005) vs. control 1.1 (0.41) mg/ml; p = 0.003) (PC_200 _Raw weanlings: RSV 0.19 (0.12) vs. control 10.2 (6.0) mg/ml MCh; p = 0.001). Increased responsiveness to aerosolised MCh was matched by elevated levels of cysLT at 5 d and elevated VEGF and PGE_2 _at 7 d in BALF from both adult and weanling mice. Responsiveness was not increased in response to iv MCh in vivo or EFS or MCh challenge in vitro. Increased epithelial permeability was not detected at 7 d.

**Conclusion:**

Infection with 1 × 10^5 ^pfu RSV induced extreme hyperresponsiveness to aerosolised MCh during the acute phase of infection in adult and weanling mice. The route-specificity of hyperresponsiveness suggests that epithelial mechanisms were important in determining the physiological effects. Inflammatory changes were dissociated from physiological changes, particularly in weanling mice.

## Introduction

Respiratory syncytial virus (RSV) infection is one of the most common diseases of childhood. It is estimated that RSV infects up to two-thirds of infants worldwide by one year of age, with almost all children infected at least once by the age of 2 [1-3]. Around 75% of children have IgG antibodies to RSV by 18 months of age [4]. Most RSV disease manifests as mild upper respiratory tract infection, however a small proportion of children go on to develop severe lower respiratory tract disease including bronchiolitis and pneumonia requiring hospitalisation. Primary infection occurs at an average age of 12 months, though the median age of infants requiring hospital admission is 2 to 3 months [5] and the highest morbidity of RSV disease is seen below the age of 6 months [6-9]. Severe cases place a large burden on the health-care system; acute bronchiolitis and bronchitis are the sixth most common causes of hospital admissions in Australian children [10]. Acute RSV lower respiratory tract infection is associated with wheezing, airways hyperresponsiveness, airflow obstruction and alterations in gas exchange (reviewed in [11]).

Mice are commonly used as experimental models of human RSV infection [12]. While inoculation with high titres of RSV is necessary for replication to occur within the lungs due to the semi-permissive nature of RSV infection in the mouse host, clinical and pathological changes vary markedly with dose. Infection with low titres (10^3 ^– 10^5 ^plaque forming units (pfu) induces peribronchial and perivascular inflammation [13-15] but fails to induce clinical signs of illness [15]. In contrast, infection with high titres of RSV (~10^7 ^pfu) induces clinical signs of illness and weight loss [15-19] in conjunction with severe histopathological changes and pneumonia [17,20,21] that can persist for long periods of time (154 days [20,21]. Current physiological data describing the effects of RSV infection are limited, particularly due to the use of the parameter 'enhanced pause' (Penh) derived from unrestrained plethysmography [20-23]. Penh is widely regarded as being primarily related to ventilatory timing and contains little information on the physiological state of the airways [24]. Few studies have examined the physiological response to bronchoconstrictor challenge in intubated mice infected with RSV [15,18,25] and the physiological alterations that occur in response to RSV are yet to be clearly defined in terms of the site of responsiveness and baseline changes in airway and parenchymal mechanics.

The aim of the present study was to assess the physiological changes occurring in the airways and parenchyma of mice infected with RSV, and to relate these alterations to the inflammatory profile induced by infection. Due to the proven success of low dose RSV models in producing inflammatory and histopathological changes, we have used a low dose (10^5 ^pfu) model of infection in order to avoid the excessive pathology and structural damage that may confound our physiological measurements. We have also sought to determine whether the physiological response to primary RSV infection differs depending on age at infection.

## Materials and methods

### Animals

BALB/c mice were selected for all studies due to their availability, level of responsiveness to bronchoconstrictor challenge and permissiveness to RSV infection [12]. Mice were obtained from the Animal Resource Centre (Murdoch, Western Australia) and maintained under specific pathogen free conditions at the Telethon Institute for Child Health Research (TICHR), with food and water available ad libitum. Experimental procedures were approved by the TICHR Animal Ethics Committee and conformed to the guidelines of the National Health and Medical Research Council of Australia.

### Infection of mice with RSV

Mice were inoculated with 1 × 10^5 ^pfu of sucrose gradient purified human RSV A2 or the equivalent concentration of sucrose buffer as weanlings (21 d; weaning) or adults (8 wk). RSV was delivered to each mouse in a 30 μl inoculum under light anaesthesia (Methoxyfluorane, Medical Developments Pty Ltd, VIC, Australia) by pipetting drops of inoculum onto one nostril until the entire volume had been aspirated. Mice were laid on their side with their mouth held closed during inoculation to prevent ingestion.

Mice were housed in individually ventilated cages (IVC Sealsafe, Tecniplast, Italy) during the acute phase of infection. Low velocity HEPA filtered air was delivered to cages maintained under negative pressure.

#### Clinical signs of illness

Mice were weighed and scored for clinical signs of illness daily until 7 d post inoculation and then every 2^nd ^or 3^rd ^day until 21 d. Mice were scored on the basis of appearance and demeanour, according to the scale described by Graham and colleagues [26]. A score of 0 indicated no visible signs of ill health; 1 – barely ruffled fur; 2 – ruffled but active; 3 – ruffled and inactive; 4 – ruffled, inactive, hunched and gaunt; 5 – dead. Mice were killed if they fell below 70% of their original bodyweight and/or had a clinical score of ≥ 3.

#### Lung viral titre

Viral titres were assessed in lung homogenates at 5 d post inoculation by TCID_50 _assay on HEp-2 cells as described in [27].

### Measurement of lung function

#### Anaesthesia

Mice were anesthetized by intraperitoneal injection of 0.1 ml/10 g bodyweight of a mixture of ketamine (40 mg/ml, Troy Laboratories, NSW, Australia) and xylazine (2 mg/ml, Troy Laboratories, NSW, Australia). No muscle relaxants were used. Two thirds of the dose was used to induce surgical anaesthesia and the remainder was given once the mouse was attached to the ventilator. Additional doses were given as required. Once surgical anaesthesia was established a tracheotomy was performed by insertion of a straight polyethylene cannula (internal diameter = 0.086 cm, length = 1.0 cm) into the distal trachea.

#### Oscillatory lung mechanics

Mice were ventilated with a *f*lexiVent^® ^small animal ventilator (SCIREQ, Montreal, PQ, Canada) at 450 breaths per minute and a tidal volume of 8 ml/kg. A positive end-expiratory pressure was set at 2 hPa. The ventilation rate was set above the normal breathing rate to suppress spontaneous breathing during measurements. Mice were allowed to stabilize on the ventilator for 5 minutes before measurements commenced. Respiratory system impedance (Zrs) was measured using a modification of the low-frequency forced oscillation technique (FOT [28] as previously described [29]. Respiratory input impedance (Zrs) was measured between 0.5 and 20 Hz by applying a composite signal containing 19 mutually prime sinusoidal waves during pauses in regular ventilation. The peak-to-peak amplitude of the oscillatory signal was 50% of tidal volume. The *f*lexiVent ventilator was used both for regular ventilation and for delivery of the oscillatory signal without the need to disturb the mice. Measurements were excluded if coherence was < 95%.

#### Constant phase parameter estimation

The constant-phase model described by Hantos et al. [30] was used to partition Zrs into components representing the mechanical properties of the airways and parenchyma. The constant-phase model [30] was fitted as follows: Zrs = R + jωI + (G-jH)/ω^α^, where R is the Newtonian resistance (primarily located in the airways but containing a contribution from the chest wall), I is the inertance, G is the co-efficient of tissue damping, H is the co-efficient of tissue elastance, ω is the angular frequency and α represents the reciprocal frequency-dependent behaviour of G & H. Strictly speaking, the parameters Raw and Iaw, respectively, include the Newtonian components of tissue resistance and tissue inertance. However, measurements in intact and open-chest rats [31,32] demonstrate that the contributions of the tissues to Raw and Iaw can be neglected. We have also previously shown that the chest wall makes little contribution to Newtonian resistance in mice and thus R ≈ Raw [33].

#### Methacholine challenge

##### i) Aerosol MCh challenge

Following measurement of baseline lung function, mice were challenged with a saline control aerosol followed by increasing concentrations of β-methacholine chloride (MCh; Sigma-Aldrich, MO, USA; 0.001 – 30 mg/ml). Aerosols were generated with an ultrasonic nebuliser (DeVilbiss UltraNeb 2000, Somerset, PA, USA) and delivered to the inspiratory line of the *f*lexiVent using a bias flow of medical air. Each aerosol was delivered for 2 minutes during which time regular ventilation was maintained. Five measurements were made at one-minute intervals following each aerosol. The peak response at each MCh dose was compared to the mean response to saline. Responsiveness is expressed as the provocative concentration of MCh required to induce a doubling of Raw or a 50% increase in G and H (PC_200 _or PC_150_). Responsiveness to aerosolized MCh was assessed at 5, 7, 10 and 21 d post RSV infection and 5 and 21 d post control inoculation in 6–10 mice per group. These days were chosen to coincide with peak viral titres, peak inflammatory response, viral clearance and resolution of lung disease, respectively [12,13].

##### ii) Intravenous MCh challenge

Intravenous MCh challenge was performed at 7 d post infection (n = 6–8 per group), the time of peak responsiveness to aerosolised MCh in both adult and weanling mice. Increasing doses of MCh were administered by constant infusion (3 – 96 μg/min/kg; Stoelting syringe pump, Wood Dale, IL, USA) via a polyethylene cannula (length = 27 cm; outer diameter = 0.061 cm) inserted into the jugular vein. MCh-induced constriction was reversed by intraperitoneal injection of atropine sulfate (120 μg or ~6 mg/kg; Pharmacia & Upjohn, WA, Australia; adapted from [34] during continued infusion of MCh at the highest rate.

#### Responsiveness of tracheal segments in vitro

Tracheal smooth muscle (TSM) responsiveness was assessed in vitro by electrical field stimulation (EFS) and MCh challenge at 7 d post infection (n = 6–7 RSV, n = 5–8 control from each age group). Mice were anaesthetised as per preparation for in vivo measurement of oscillatory mechanics. Tracheal segments of approximately 0.5 cm in length were removed and cleaned of loose connective tissue and placed in 50 ml organ baths (Radnotti Glass Technology, CA, USA). The TSM segment was attached to a fixed lower support and a tri-shape tissue support connected to a force-displacement transducer (Model FT03E; Grass Instrument Co., MA, USA). The tissue was suspended between horizontal platinum wire electrodes (AD Instruments, NSW, Australia).

The tissues were bathed in modified Krebs-Henseleit solution containing (in mM): 118NaCl, 25NaHCO_3_, 2.8CaCl_2_.2H_2_O, 1.17MgSO_4_, 4.7 KCl, 1.2KH_2_PO_4 _and 11.1 glucose. The baths were aerated with a 95% O_2_-5% CO_2 _gas mixture. The temperature of the baths was maintained at 37°C. Each TSM segment was equilibrated in the bath for 30 min at an optimal resting tension of 0.70 g. During this equilibration time, the tissue was challenged once with 10^-4 ^M MCh. Tissues that did not develop a contractile response were excluded from further studies. Tissues were rinsed with fresh Krebs-Henseleit solution periodically and allowed to relax to their initial tension after reaching maximal contraction.

Recordings of resting tensions and TSM contractile responses were made using a PowerLab 8/s Recorder and Chart 5.1.1 software (AD Instruments, NSW, Australia). EFS (30 V, 3 ms square wave pulses at 0.5, 1, 2, 5, 10, 20, 30, 40 Hz) were delivered via platinum electrodes by a Grass S44 stimulator connected to a stimulus isolation unit (Grass Instruments, MA, USA). The stimulus was applied until the tissue reached a maximum contraction (~10 s). The tissue was washed after every second stimulation to ensure that the relative concentrations of the ions in the Krebs-Henseleit solution were maintained. EFS responsiveness is expressed as the frequency required to induce 50% of the maximal contractile response (EC_50_). To assess cholinergic sensitivity of the tissues, cumulative dose-response curves to MCh were performed in half-log increments employing concentrations ranging from 10^-8 ^to 10^-4 ^M. Results from MCh challenge are expressed as a percentage of the maximal contractile response as well as the EC_50_. Tissues were washed and rested repeatedly between EFS and MCh challenge.

### Bronchoalveolar lavage and lung fixation

Lungs were lavaged at the completion of lung function measurements and just prior to death of the animal by washing 1 ml of ice-cold lavage fluid (0.9% saline containing 0.35% lidocaine (Sigma, St Louis, MO, USA) and 0.2 % BSA (CSL Ltd, Parkville, VIC, Australia) in and out of the lungs three times. Bronchoalveolar lavage fluid (BALF) was processed for total and differential cell counts. Cytospins for differential counts were stained with Leishmans stain (BDH Laboratory Supplies, Poole, England). Lavage supernatants were stored at -80°C. Total and differential cell counts were performed on lavage samples from 6–10 mice per group.

Lungs were inflation fixed in situ in 10% phosphate-buffered formalin (Confix, Autralian Biostain Pty Ltd, VIC, Australia) at a distending pressure of 10 hPa for 1–2 hours before ligation and removal from the chest cavity. Lungs were immersion fixed in formalin overnight before being transferred to 70% ethanol and stored at 4°C until processing. Paraffin embedded lungs were sectioned at 5 μm thickness and stained with haematoxylin and eosin.

#### Measurement of cytokines and mediators in BALF

In order to characterise the primary inflammatory and cytokine response to RSV infection, we chose the appropriate kit to measure innate immune responses. This included tumour necrosis factor alpha (TNFα), interferon gamma (IFNγ), macrophage chemotactic protein 1 (MCP-1) and interleukins (IL) 6, 10 and 12 (p70 protein) and these were measured in BALF supernatants by cytometric bead assay (BD Biosciences, CA, USA) according to the manufacturer's instructions. Prostaglandin E_2 _(PGE_2_), IL-13, vascular endothelial growth factor (VEGF) and cysteinyl leukotrienes (cysLT) were measured as potential mediators of airway hyperresponsiveness using enzyme immunoassay kits (PGE_2_, cysLT: Cayman Chemicals, MI, USA; IL-13, VEGF: Quantikine, R&D Systems, MN, USA) according the manufacturer's instructions. Cytometric bead assay and cysLT ELISA were performed at 5, 7 and 21 d post RSV inoculation and at 5 and 21 d post diluent control inoculation. IL-13, VEGF and PGE_2 _were measured at 5 and 7 d post RSV inoculation and at 5 d post control inoculation.

### Measurement of epithelial permeability using Evans Blue dye

Evans Blue dye (EBD) is a useful indicator of microvascular permeability [35]. EBD (Sigma-Aldrich, MO, USA) was administered intravenously to mice via the jugular vein following iv MCh challenge as described by Tulic et al. [36]. A slow bolus of 50 mg/kg EBD was delivered in a volume of 0.1 ml/10 g bodyweight through the existing iv cannula. Mice were ventilated for a further 30 minutes before post-EBD BAL was performed. The amount of EBD in BALF was quantified by reading the absorbance of the samples at 620 nm using a microplate reader (Bio-Tek Instruments, VT, USA). The amount of dye was calculated by interpolation on a standard curve in the range of 1 – 10 μg/ml [37]. Measurement of epithelial permeability was performed at 7 d post infection in adult mice only (n = 8 control, 7 RSV).

### Statistical analysis

RSV groups were compared vs. combined control groups where no differences were observed between controls at 5 and 21 d. Differences in bodyweight, viral titre and EBD concentrations between groups were compared using unpaired t-test. Differences in total and differential cell counts, baseline physiology, cytokine and mediator assays were tested by 1-way analysis of variance (ANOVA) followed by Dunnett's post-hoc test for normally distributed data, and by Kruskal-Wallis ANOVA on ranks followed by Dunn's test for non normal data. Differences in MCh responsiveness in vivo between RSV infected and control animals were tested by 1-way ANOVA on PC_200/150 _data for aerosol MCh challenge, and by 2-way repeated measures ANOVA for iv MCh challenge. In vitro responsiveness of TSM segments was tested using 1-way ANOVA on EC_50 _data. Data are expressed as mean (SE). Graphs were prepared using SigmaPlot software (SigmaPlot 2000, SPSS Science, IL, USA). Statistical analysis was performed using SigmaStat software (version 2.03, SPSS Science, IL, USA). Significance was accepted at p < 0.05.

## Results

### Clinical illness

**Figure 1:**
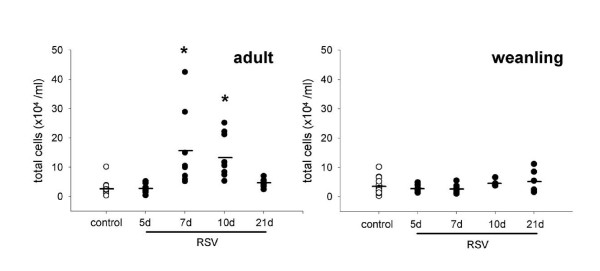
Total cells in BALF from adult and weanling mice inoculated with RSV or diluent control. Adult mice had significantly elevated total cell numbers in BALF at 7 and 10 d post inoculation that returned to control levels by 21 d. Weanling mice did not have increased cell numbers in BALF at any timepoint.

Mice infected with RSV did not exhibit clinical signs of illness during the acute phase of infection. Adult mice infected with RSV did not decrease in bodyweight compared to controls (p = 0.41). RSV infected weanling mice gained weight at the same rate as control animals, both groups reaching 125–130% of their original bodyweight by 5 d post inoculation (p = 0.66; Figure 1). No mice were culled for excessive weight loss or clinical score ≥ 3.

### Viral titre

Adult and weanling mice had similar levels of RSV replication in lung homogenates at 5 d post inoculation (4.96 and 4.92 × 10^4 ^TCID_50_/g, respectively).

### Inflammation

#### Adult mice

**Figure 2:**
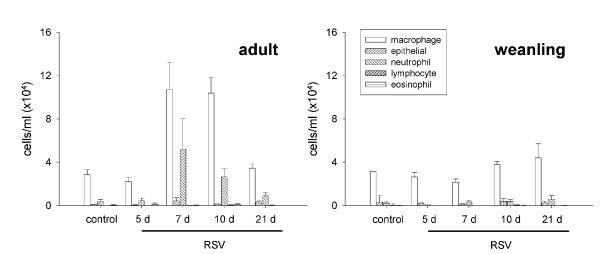
Differential cell counts in adult and weanling mice after RSV and control inoculation. Macrophages were the predominant cell type in both age groups. Total macrophage and neutrophil numbers were increased in adult mice at 7 and 10 d post infection; however this did not reach statistical significance.

**Figure 3:**
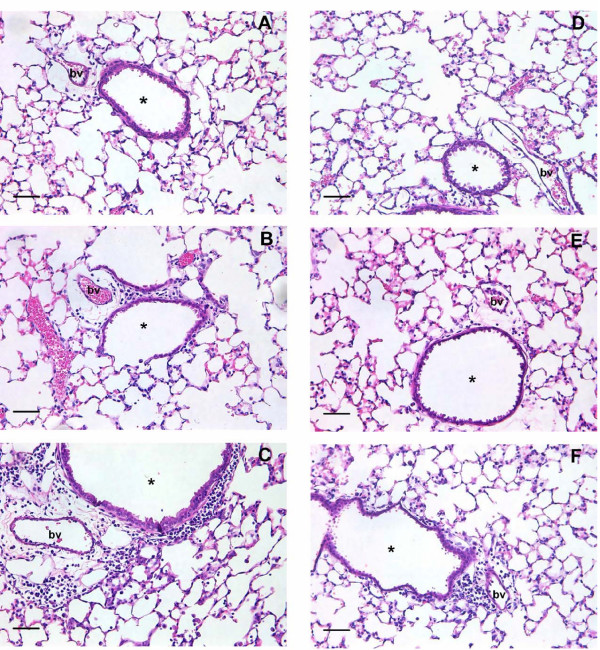
Representative sections from adult (A-C) and weanling (D-F) mice inoculated with diluent control (A, D) or RSV (B, C, E, F), each showing an airway (*) and blood vessel (bv). Perivascular and peribronchiolar inflammation were evident to a small degree at 5 d post RSV (B); and to a much greater extent at 7 d post RSV (C) in adult mice. Some parenchymal inflammation was also present at 7 d. Little to no evidence of inflammation existed in weanling mice at 5 d post infection (E); however a small degree of perivascular and peribronchiolar inflammation was present at 7 d post infection (F). Based on morphology these cells were classified as lymphocytes. Control mice did not show any evidence of inflammation at either age (A, D). Bar = 50 μm.

Adult mice had significantly increased inflammatory cell numbers in BALF at 7 and 10 d post inoculation (p < 0.001). Cell numbers had returned to control levels by 21 d (Figure 1). Despite increased cell numbers, differential cell counts did not reveal a difference in the type of infiltrating cells at any timepoint and were dominated by macrophages (Figure 2). Mild peribronchiolar and perivascular inflammation was evident in histological sections at 5 d post RSV infection (Figure 3B), and had increased in severity at 7 d post infection (Figure 3C). Inflammatory cells were also visible in the lung parenchyma at 7 d (Figure 3C). Control mice did not show any evidence of inflammation at 5 d post inoculation (Figure 3A).

#### Weanling mice

Inflammatory cell numbers in BALF did not change in weanling mice inoculated with RSV or diluent control (p = 0.191; Figure 1). Similarly, there was no difference in cell profile in BALF (Figure 2). Histological sections from weanling mice inoculated with diluent control and at 5 d post RSV infection showed little or no inflammatory infiltrate around airways, blood vessels or in the lung parenchyma (Figure 3D, E, respectively). Peribronchiolar and perivascular inflammation were evident to a small extent at 7 d post infection (Figure 3F), with infiltration of lymphocytes seen.

### Airway and parenchymal mechanics

#### Baseline lung function

In keeping with the mild inflammatory changes observed in histological sections, there was no evidence of airway obstruction or increased tissue stiffness at baseline in RSV-infected mice. RSV infection did not alter baseline Raw, G or H in adult mice (Table 1). Weanling mice had higher values of Raw, G and H than adult animals, consistent with age-related alterations in respiratory mechanics [38], although H decreased to approach adult values by 21 d (Table 1). Raw and G were not altered in RSV-infected weanling mice at baseline. H was decreased in weanling mice at 21 d post infection, but only when compared to 5 d controls (p = 0.003; p < 0.05 vs. 5 d control).

**Table 1:** Baseline airway and tissue mechanics in adult and weanling mice. Values: mean (SE).

**Age**	**Treatment**	**Weight (g)**	**Raw hPa.s.ml**^**-1**^	**G hPa.ml**^**-1**^	**H hPa.ml**^**-1**^
Adult	Control 5 d	19.3 (0.4)	0.33 (0.02)	5.1 (0.2)	37.3 (1.3)
	Control 21 d	17.9 (0.6)	0.33 (0.02)	5.4 (0.5)	36.5 (2.3)
	RSV 5 d	17.1 (0.2)	0.38 (0.03)	5.2 (0.2)	40.9 (1.8)
	RSV 7 d	18.2 (0.3)	0.35 (0.03)	5.9 (0.3)	44.3 (2.4)
	RSV 10 d	16.7 (0.3)	0.39 (0.03)	5.2 (0.3)	41.5 (2.1)
	RSV 21 d	18.7 (0.4)	0.43 (0.02)	4.8 (0.3)	40.3 (2.5)
Weanling	Control 5 d	13.9 (0.6)	0.51 (0.04)	7.0 (0.4)	61.6 (2.6)
	Control 21 d	16.7 (0.6)	0.48 (0.03)	6.5 (0.8)	57.7 (2.9)
	RSV 5 d	13.9 (0.5)	0.53 (0.08)	7.6 (0.4)	69.6 (5.7)
	RSV 7 d	15.1 (0.3)	0.52 (0.03)	7.8 (0.5)	64.0 (3.1)
	RSV 10 d	15.5 (0.5)	0.52 (0.05)	8.5 (0.7)	65.6 (6.7)
	RSV 21 d	16.3 (0.5)	0.39 (0.02)	6.3 (0.4)	45.1 (3.5)*

* p < 0.05 vs. d5 control, not significant vs. d21 control

#### Responsiveness to MCh

##### i) Aerosol MCh challenge

**Figure 4:**
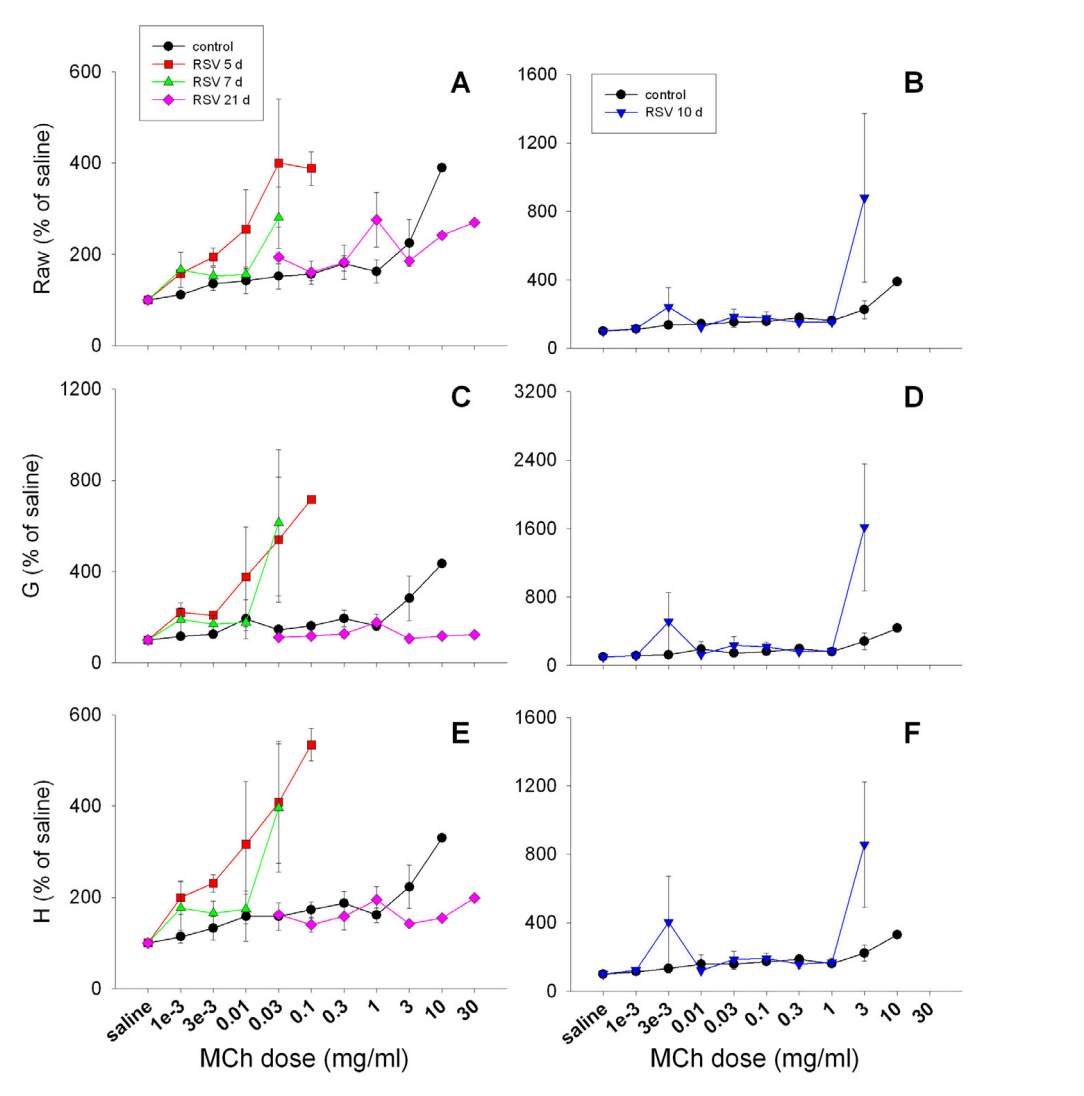
Dose-response curves to aerosolised MCh challenge in adult mice showing airway resistance (A, B), tissue damping (C, D) and tissue elastance (E, F). Hyperresponsiveness was clearly evident in airways and tissues at 5 and 7 d post RSV infection (A, C, E). A mixed response was seen at 10 d post infection (B, D, and F).

**Figure 5:**
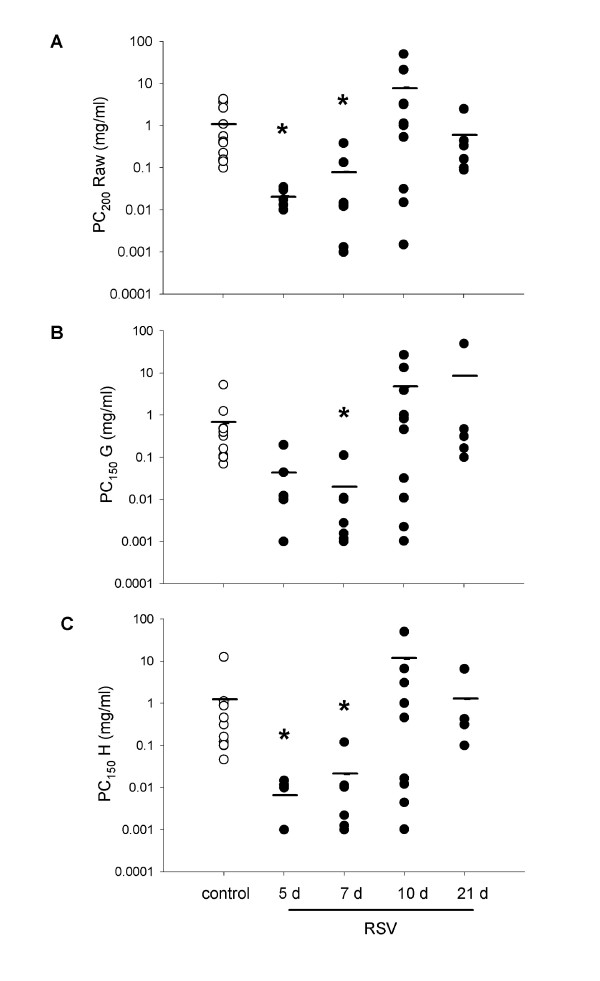
Concentrations of aerosolised MCh required to induce a doubling of the response to saline in the airways (A), or a 50% increase in the response of the lung parenchyma (B, C) of adult mice. Significantly lower concentrations of MCh were required to induce responses at 5 and 7 d post infection in Raw and H (A, C), and at 7 d in G (B). A mixed response was evident at 10 d post infection in both airway and tissue compartments.

Adult mice exhibited extreme hyperresponsiveness to aerosolised MCh (Figure 4) in both airway and tissue compartments at 5 and 7 d post RSV inoculation (Raw, G, H: p = 0.003, 0.007, <0.001, respectively), requiring an approximately 100-fold lower concentration of MCh than control animals to elicit a doubling of the response (Figure 5). The response to MCh at 10 d was more variable, with approximately half of the mice studied having returned to control levels of responsiveness by this timepoint. Responsiveness had returned to control levels in all animals studied by 21 d.

**Figure 6:**
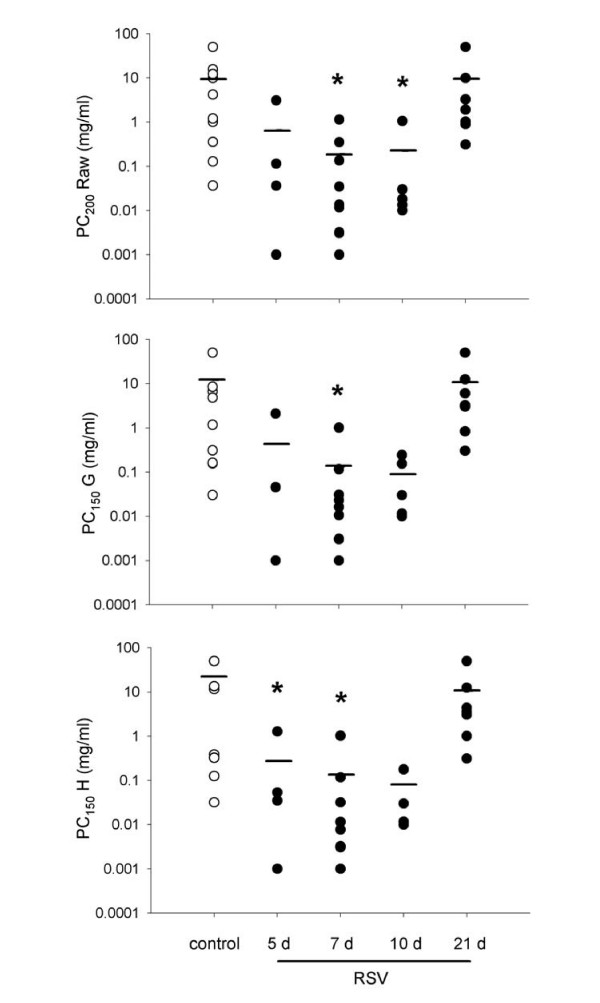
Concentrations of aerosolised MCh required to induce a doubling of the saline response in the airways (A), or a 50% increase in the response of the lung parenchyma (B, C) of weanling mice. Significantly lower concentrations of MCh were required to induce responses at 7 and 10 d post infection in Raw (A), 7 d in G (B) and 5 and 7 d in H (C). The response at 10 d post infection was more consistent than in adult mice, however responsiveness in general was much more variable in weanlings.

Weanling mice had more variable responses to MCh but were still hyperresponsive to aerosolised MCh at 5 and 7 d (p = 0.001, < 0.001, <0.001 for Raw, G, H respectively) (Figure 6). A mixed response was again seen at 10 d. Weanling mice required an approximately 10-fold lower concentration of MCh to elicit a response. Responsiveness had returned to control levels by 21 d.

##### ii) Intravenous MCh challenge

**Figure 7:**
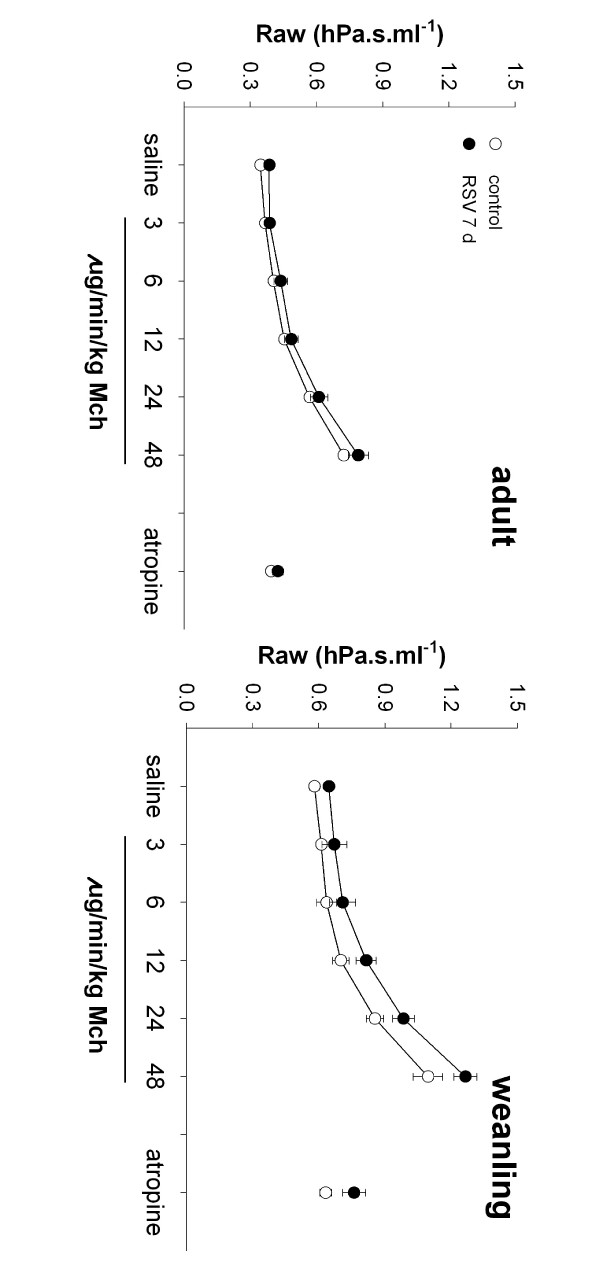
Airway resistance in response to iv MCh challenge in adult and weanling mice at 7 d post infection. RSV infected mice (closed symbols) did not demonstrate increased responsiveness to any concentration of iv MCh compared to controls (open symbols) at either age. Raw returned to baseline levels following atropine administration. RSV-infected weanling mice had slightly elevated Raw throughout the iv challenge, although this was due to their smaller size rather than altered responsiveness.

Neither adult nor weanling mice exhibited increased airway or tissue responsiveness to iv MCh compared to controls at 7 d post inoculation. Weanling mice infected with RSV were slightly smaller than controls (control 13.7 (0.35) g; RSV 11.8 (0.35) g, causing a small upward shift in the curve that was not related to altered responsiveness (Figure 7). This weight difference was maintained from the time of inoculation and due to variation in litter size rather than weight loss from RSV-induced illness.

#### In vitro responsiveness

**Figure 8:**
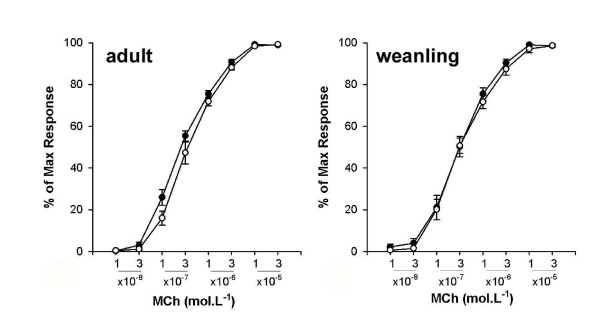
Responsiveness of isolated TSM segments from adult and weanling mice to MCh challenge at 7 d post infection. RSV infected mice (closed symbols) did not demonstrate altered bronchoconstrictor responses in vitro compared to controls (open symbols) when infected as either adults or weanlings.

TSM segments from adult and weanling mice infected with RSV did not exhibit increased responsiveness to EFS post inoculation (adult EC_50 _(Hz): RSV 2.59 (1.32) vs control 1.68 (0.56); weanling EC_50 _(Hz) RSV 2.23 (0.74) vs control 1.77(0.84). Similarly, there was no change in responsiveness to MCh at 7 d (Figure 8).

### Cytokines and mediators in BALF

#### Cytometric bead assay

**Figure 9:**
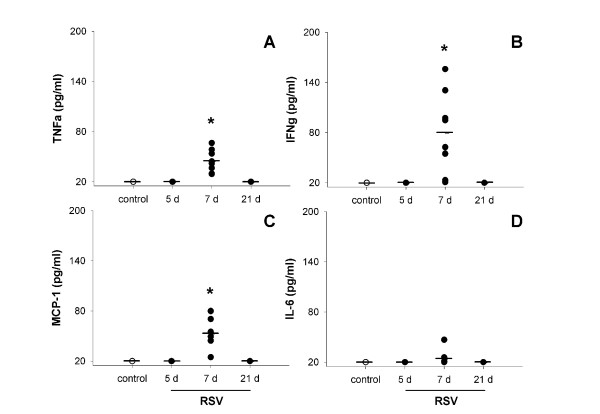
Cytokine levels in BALF from adult mice. TNFα (A), IFNγ (B) and MCP-1 (C) concentrations were all increased at 7 d post infection and undetectable in controls and at other timepoints. IL-6 was elevated to a small extent at 7 d (D) but did not reach significance in post-hoc analysis.

IL-12 p70 was not detectable in BALF from adult mice at any timepoint, irrespective of treatment (data not shown). TNFα, IFNγ, MCP-1 and IL-6 were undetectable in control samples and at 5 and 21 d post RSV inoculation but were significantly increased at 7 d post RSV (p < 0.001; Figure 9A–C). IL-6 was increased at 7 d but did not reach significance in post-hoc analysis (p = 0.011; Figure 9D). IL-10 levels were not altered by RSV infection (p = 0.125, data not shown).

IL-12 p70, TNFα, IFNγ, MCP-1 and IL-6 were all below detectable levels in BALF from weanling mice at all timepoints (data not shown). Although detectable, IL-10 levels were not altered by RSV infection (data not shown).

#### Prostaglandin E_2_

**Figure 10:**
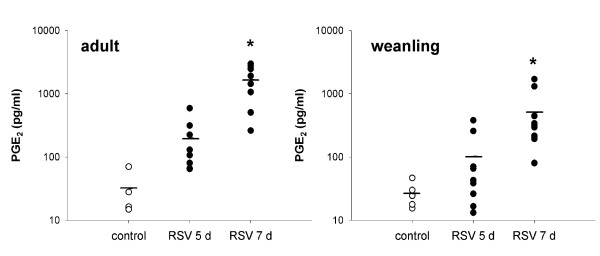
PGE_2 _levels in BALF from adult and weanling mice. Significantly elevated levels were detected at 7 d post RSV infection in both age groups.

PGE_2 _was elevated in BALF from both adult and weanling mice, peaking at 7 d post infection (p < 0.001) (Figure 10).

#### Cysteinyl leukotrienes

**Figure 11:**
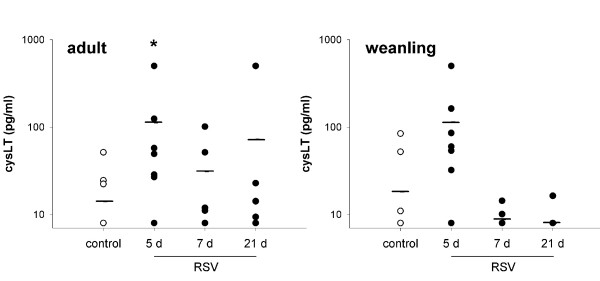
cysLT concentrations in BALF from adult and weanling mice. Elevated levels were detected at 5 d post RSV infection in adults and weanlings, although this did not reach significance in post-hoc analysis in weanlings. Upper and lower limits of detection for this assay were 8 and 500 pg/ml, respectively.

Increased levels of cysLT were detected in BALF from adult and weanling mice (p = 0.029, 0.009 respectively), peaking at 5 d post RSV inoculation (Figure 11). Despite a great deal of variability, the increase was significant in adult mice at 5 d (p < 0.05), but did not reach significance in weanling mice.

#### IL-13

IL-13 was undetectable in all samples (data not shown).

#### VEGF

**Figure 12:**
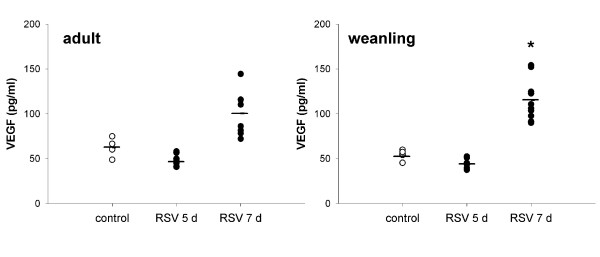
VEGF levels in BALF from adult and weanling mice. Elevated levels were detected at 7 d post RSV infection.

VEGF was elevated at 7 d post infection in both adult and weanling mice (both p < 0.001). Neither age group had elevated VEGF levels at 5 d post infection (Figure 12).

### Microvascular permeability

Microvascular permeability measured by Evans blue dye extravasation into BALF was not increased in adult mice at 7 d (p = 0.25; data not shown). Microvascular permeability was not measured in weanling mice.

## Discussion

Our low dose model of RSV infection was successful in achieving viral replication and physiological alterations to airway (Raw) and parenchymal (G, H) function in the lungs of both adult and weanling mice. The level of responsiveness was somewhat dissociated from the observed inflammatory changes, particularly in the younger mice. As expected with the dose of RSV administered [15], mice did not lose weight or show clinical signs of illness during the acute phase of infection. Weanling mice infected with RSV gained weight at the same rate as controls.

### Inflammatory changes

Adult mice showed modestly elevated inflammatory cell numbers in BALF that peaked at 7 d post infection and returned to control levels by 21 d (Figure 1). Inflammatory cell numbers were not elevated in adult mice at 5 d post infection, indicative of a delay between viral replication in the lung and initiation of the cell-mediated immune response. Weanling mice did not show any increase in infiltrating inflammatory cell numbers above control mice at any timepoint, suggesting that this level of viral replication was insufficient to induce a detectable cell-mediated immune response. The cell profile was not altered in either age group, and consisted predominantly of macrophages at all timepoints (Figure 2). Histological sections revealed a mild peribronchiolar and perivascular inflammation 5 d post infection in adult mice, becoming more extensive at 7 d. Weanling mice at 5 d did not appear different to controls, however a small degree of inflammation was seen in weanling mice at 7 d post infection (Figure 3).

The cytokine profile measured in BALF from adult and weanling mice mirrored the inflammatory profile, with adult mice showing increased levels of TNFα, IFNγ, MCP-1, IL-6 and VEGF at 7 d post infection but not at 5 d (not measured at 10 d) (Figures 9 and 12). In keeping with inflammatory cell numbers, weanling mice did not have elevated TNFα, IFNγ, MCP-1 or IL-6 in BALF at any timepoint, although VEGF was elevated at 7 d post infection (Figure 12). IL-13 has previously been identified as an important mediator of AHR in RSV infected DBA/J and BALB/c mice [25], however it was undetectable in the present study. In the absence of significant numbers of infiltrating lymphocytes, the absence of IL-13 in these samples is unsurprising. These results suggest that the physiological changes observed in RSV infected mice in the present study were not due to cell-mediated inflammatory processes.

### Physiological changes

#### Baseline physiology

RSV infection did not alter baseline airway or parenchymal mechanics in mice infected as adults or weanlings (Table 1). Age-related differences in respiratory mechanics were apparent between age groups and in weanling mice between the 5 and 21 d timepoints, although this pattern was not altered by RSV infection. These data suggest that the low levels of inflammation observed in tissue sections were insufficient to cause airway obstruction (increased Raw) or stiffening of parenchymal tissues (increased G and/or H).

#### Aerosol MCh

Both adult and weanling mice demonstrated extreme airway and parenchymal hyperresponsiveness to aerosolised MCh, although the response in weanlings was more variable. Adult mice required an approximately 100-fold lower dose of MCh than controls for a doubling response (Figures 4, 5); weanlings required on average 10-fold less MCh than control animals (Figure 6). The concentration of MCh required for response seen in these mice is substantially lower than has been demonstrated by other studies (generally requiring ~10 mg/ml) using similar infective doses of RSV [22,23,39,40]. Control mice responded at a somewhat lower MCh dose than naïve BALB/c routinely measured in our laboratory (data not shown), indicating that the sucrose buffer solution may have induced some degree of hyperresponsiveness. Despite the level of responsiveness of the control animals, RSV infection still induced a clear leftward shift of both the airway and parenchymal dose-response curves representing increased sensitivity to bronchoconstrictor challenge.

AHR to MCh in RSV-infected mice has primarily been detected using unrestrained plethysmography [22,23,39], an inherently non-specific means of measuring airway function [24]. We were not surprised at differences between our results and those obtained with Penh, given that FOT contains direct physiological information on airway and parenchymal behaviour. Penh data obtained during MCh challenge is also likely to be contaminated by increased nasal resistance due to respiratory secretions induced by cholinergic stimulation. A potential advantage of unrestrained plethysmography over FOT may lie in the ability to test unsedated animals, but we would expect sedation to reduce responsiveness rather than increase it [41,42]. More reliable physiological data comes from Dakhama et al. [40], who demonstrated AHR to MCh in intubated mice using total lung resistance. The magnitude of the response detected in the present study coupled with the similar pattern of responsiveness in the airway and parenchymal compartments suggests that degree of sensitivity to MCh detected in the present study is not simply a function of a more sensitive measurement technique relative to other studies. While partitioning of Zrs into airway and tissue mechanics allows detection of more subtle changes than total lung or respiratory system impedance, it seems unlikely that responses of this magnitude would not be detected using other measurement systems.

#### Intravenous MCh and MCh in vitro

In contrast to challenge with aerosolised MCh, responsiveness to iv MCh challenge was not altered in mice infected with RSV as adults or weanlings (Figure 7). Similarly, we did not observe increased responsiveness to MCh in tracheal segments from adult or weanling mice at 7 d (Figure 8). The contrasting effects of different routes of agonist delivery suggest that delivery of MCh directly to the epithelial surface is crucial in inducing hyperresponsiveness. Conflicting data exists in the literature on the response to iv MCh in RSV-infected mice; similar doses of iv MCh have been shown to induce hyperresponsiveness in mice infected with 3 × 10^5 ^pfu RSV [25] and 10^7 ^pfu RSV [15], and to be unable to induce hyperresponsiveness in mice infected with 10^7 ^pfu RSV [18].

#### Responsiveness to EFS in vitro

In the present study, responsiveness of tracheal segments to EFS in vitro was unaltered in RSV-infected mice. Using a slightly higher dose of RSV (10^6 ^pfu), Dakhama et al. [40] demonstrated increased responsiveness of murine tracheal smooth muscle segments to EFS at 6 d post infection, without any increase in maximal tension. Similarly, Colasurdo and colleagues have demonstrated increased responsiveness to EFS but no change in maximal tension at 4 d post infection in cotton rats [43]. The reason for the discrepancy in EFS responsiveness between published studies and the results we report here is not clear. While the dose used by Dakhama et al. [40] was somewhat higher, inflammatory cell numbers in BALF were identical (in adult mice) to our study, suggesting similar severity of infection. Differences in responsiveness between mice and cotton rats does not seem surprising given the greater permissiveness of cotton rats to RSV infection, however the similar pattern of responses seen in the aforementioned studies suggests that species differences do not play a major role. The lack of an increased responsiveness to EFS *in vitro *does, however, argue against any alterations in neural control of airway smooth muscle (ASM) and the lack of increased responsiveness to MCh *in vitro *argues against alterations in ASM contractile properties following RSV infection.

### Mechanisms of route-specific hyperresponsiveness

The discrepancy in responsiveness between in vivo and in vitro conditions may reflect differences in the site of responsiveness within the airway tree. The use of extrathoracic tracheal segments in vitro may ignore alterations in airway function occurring further down the airway tree. This may be particularly relevant when the site of RSV replication within the mouse lung is considered; the virus replicates mostly in small airways and alveolar epithelial cells rather than epithelial cells in large conducting airways. While technically more difficult, assessment of the responsiveness of bronchi or intrathoracic airways may yield a greater response in vitro [44]. Airway resistance as measured in vivo represents a greater proportion of the airway tree and thus may be more sensitive to changes occurring in regions of the airway other than the trachea.

Differences in levels of responsiveness between different modes of agonist delivery in the present study bear strong resemblance to studies investigating the effects of cationic proteins on airway function published in the 1990's [45-47]. After the discovery that treatment with cationic proteins (major basic protein, poly-L-lysine and poly-L-arginine) increased airway responsiveness to inhaled MCh in rats [47], further investigation in isolated airways revealed that hyperresponsiveness was induced only if MCh was delivered to the luminal surface of the airway in vitro. Delivery of MCh to the external surface of the airway wall did not alter responsiveness irrespective of pre-treatment with cationic proteins [45,46]. Thus, hyperresponsiveness was only manifested when the challenging agonist had to cross the airway epithelium to reach the underlying ASM, and implicates the integrity of the epithelial layer as important in the response to inhaled agonists.

Epithelial mechanisms are the most likely candidates to explain the results of the present study, in which hyperresponsiveness was only induced by aerosol delivery of MCh in vivo. We did not examine luminal vs. external administration of MCh in vitro, but can instead compare aerosol and iv delivery in vivo. Disruption of epithelial barrier function or alterations in signalling are potential candidates to explain the site-specificity of agonist action of airway responsiveness. VEGF is upregulated in nasal washings from RSV-infected children [48] and in human epithelial cells infected with RSV in vitro [48,49]. VEGF was found to be responsible for increased permeability of RSV-infected epithelial monolayers in culture [49]. In the present study, VEGF was detected at elevated levels in BALF of both adult and weanling mice at 7 d post infection but was not elevated at 5 d (Figure 12), suggesting that it may play a role in increasing airway responsiveness but cannot account for increased responsiveness at all timepoints. Despite elevated levels of VEGF, and contrary to the results of Kilani et al. in vitro [49], epithelial permeability as measured by Evans Blue dye extravasation into BALF was not increased at the time of peak responsiveness (7 d post infection) in RSV infected mice in vivo. These results suggest that epithelial barrier function remained intact in these animals; however the level of permeability of the epithelium in these mice simply may not have been great enough to allow extravasation of the large Evans Blue dye-albumin complex whilst being sufficient to allow greater access of MCh (~300 × smaller than albumin) to the ASM. Alternatively, the ability of the technique to detect small increases in epithelial permeability may have been limited by binding of Evans Blue dye to airway tissues [50].

Surfactant acts as part of the airway mucosal barrier and may be involved in the epithelial-specific response to MCh challenge observed in the present study. Multiple layers of phospholipid bind directly to the surface of the bronchial epithelium (reviewed in [51] and contribute to epithelial barrier function by masking bronchial irritant receptors that respond to MCh challenge [52,53]. Disruption of surfactant function by RSV infection (as demonstrated in mice by [54] and unmasking of irritant receptors may be sufficient to enhance the level of responsiveness to aerosolised MCh, although we have not performed any studies to directly test this hypothesis. Alterations in responsiveness induced by surfactant dysfunction in RSV infection would only be detected by MCh delivery directly to the epithelial surface and would not require increased epithelial permeability. Bypassing the epithelial layer by iv administration of MCh and the loss of the surfactant layer in vitro would not reveal disruption of surfactant function, and are supported by the results of the present study.

### Role of cytokines and mediators in AHR

Airway and parenchymal hyperresponsiveness were dissociated from inflammatory changes in the low dose model of RSV infection used in the present study. The level of hyperresponsiveness seen in these mice far exceeded that which would be expected for the measured levels of inflammation. The dissociation between inflammatory and physiological changes is further emphasised by the similarity of physiological responses between adult and weanling mice despite their differing inflammatory profiles. In the absence of a significant population of inflammatory cells, the innate immune response and products of resident cells become potential candidates for induction of hyperresponsiveness. The potential roles of leukotrienes and prostaglandins in the hyperresponsiveness seen in RSV-infected mice in the present study were investigated due to their ability to influence ASM contraction.

Cysteinyl leukotrienes have profound effects on airway function; they are potent activators of ASM contraction [55], they act on the vasculature to produce vasodilation and increase vascular permeability [56] and stimulate mucus secretion and interfere with mucociliary clearance [57]. The role of PGE_2 _in regulating airway function is more complex, due in part to the existence of four separate cell surface receptors with unique signal transduction mechanisms [58]. PGE_2 _is potently bronchoprotective in vitro (reviewed in [59], but has been shown to induce bronchoconstriction as well as bronchoprotection in humans [60,61] and animal models [62,63]. Although the distribution of PGE_2 _receptors within the lung has not been fully defined, mRNA expression of all four types has been detected in the mouse lung [64,65]. Airway epithelial cells, mast cells and alveolar macrophages are local sources of cysLT and PGE_2_, both of which have been shown to be elevated in the airways of children with bronchiolitis [66-68].

Cysteinyl leukotrienes were significantly elevated in both adult and weanling mice infected with RSV prior to influx of inflammatory cells at 7 d post infection (Figure 11), suggesting that epithelial cells were the main source. cysLT expression was detected earlier than PGE_2_, peaking at 5 d post infection in adult mice, and only elevated at 5 d in weanling mice. Although consistently detectable at 5 d post RSV in both age groups, cysLT levels varied markedly between animals. PGE_2 _was detected at significantly elevated levels in BALF from both adult and weanling mice at 7 d post infection (Figure 10). The similarity between adult and weanling PGE_2 _levels despite the dramatically different infiltrating cell populations at these two ages again suggests that epithelial cells were the predominant source. The extreme sensitivity of both adult and weanling mice to MCh challenge at the peak of PGE_2 _production indicates that the bronchoprotective effect of PGE_2 _at these concentrations was not sufficient to inhibit airway responsiveness.

Coyle et al (JCI 1995) demonstrated that the cationic proteins major basic protein and poly-L-lysine increased immunoreactive kinins and kallikrein-like activity *in vivo *and that this mechanism explained the epithelial-dependant increase in MCh responsiveness. We did not have the opportunity of studying kinins and so can not comment on whether similar mechanisms may underlie the epithelial-dependent MCh responsiveness we report following RSV infection.

### Age-dependent effects of RSV infection

Despite equal levels of viral replication in adult and weanling mice, significant differences were observed in the inflammatory response to RSV. The lack of a significant cell-mediated immune response in weanling mice suggests that differences in the level of the host innate immune response to RSV may have been responsible for the disparity in responsiveness between the two age groups. The lack of MCP-1 expression detected in BALF from weanling mice may be indicative of a general paucity of chemoattractant chemokine production in this age group. The similarity of physiological responses despite marked differences in cell mediated immunity between adult and weanling mice highlights the issues associated with characterising infection models solely in adult animals with mature immune systems. These data also argue for the need for a systematic study of the effect of age on the effects of viral infections in mouse models.

### Summary

Infection of adult and weanling mice with 1 × 10^5 ^pfu RSV induced significant alterations in airway and parenchymal responsiveness to bronchoconstrictor challenge. Increased responsiveness occurred in the absence of baseline changes in airway or parenchymal physiology, and in conjunction with mild inflammatory changes. Route-specificity of MCh responsiveness and elevated levels of epithelial-derived mediators indicated that epithelial mechanisms were the main determinants of altered respiratory function.

The model described in the present study may provide a useful basis for assessment of the specific physiological effects of mild RSV lower respiratory tract infection on airway function. Although great caution should always be maintained when translating data from mouse models to humans, VEGF-mediated increases in epithelial permeability may be a mechanism by which RSV mediates airways hyperresponsiveness in the human disease.
